# Self-Directed Learning and Competencies as Perceived by New Graduates Before and After the Pandemic: A Repeated Cross-Sectional Study

**DOI:** 10.1155/jonm/1756024

**Published:** 2025-05-27

**Authors:** Sara Dentice, Alessandro Galazzi, Stefania Chiappinotto, Satu Kajander-Unkuri, Luca Grassetti, Anna Brugnolli, Alvisa Palese

**Affiliations:** ^1^Department of Medicine, University of Udine, Udine, Italy; ^2^Italian National Institute of Health, Rome, Italy; ^3^Università LUM Giuseppe Degennaro, Bari, Italy; ^4^Department of Nursing Science, Faculty of Medicine, University of Turku, Turku, Finland; ^5^The Health Sciences Unit, Faculty of Social Sciences, Tampere University, Tampere, Finland; ^6^Department of Economics and Statistics, University of Udine, Udine, Italy; ^7^Interdepartmental Medical Science Centre, University of Trento, Trento, Italy

**Keywords:** competencies, COVID-19, new graduates, pandemic, self-directed learning skills

## Abstract

**Background:** The COVID-19 pandemic led to significant changes in nursing education; however, their impact on competencies and self-directed learning (SDL) skills achieved at the point of graduation has been limitedly investigated.

**Aims:** To compare the perceived SDL skills and competencies at the time of graduation between pre- and postpandemic graduates; and to assess correlations, if any, between the SDL skills and competencies in both groups.

**Design:** A repeated cross-sectional study design following the Strengthening of the Reporting of Observational Studies in Epidemiology checklist.

**Methods:** All 2019 (prepandemic) and 2023 (postpandemic group) new graduates from two Italian Universities and willing to participate were included. The Self-Rating Scale of SDL (SRSSDL_ITA_) and the Nurse Competence Scale (NCS) were administered. Descriptive and inferential statistics were used.

**Results:** Postpandemic graduates reported slightly lower SDL scores (pre- 4.27 vs. postpandemic 4.18 out of 5, *p* = 0.030), with significant declines in “Awareness” (*p* = 0.005), “Learning Strategies” (*p* < 0.001), and “Interpersonal Skills” factors (*p* = 0.007). Perceived overall competence as measured with the NCS was higher in the postpandemic group (pre- 68.01 out of 100 vs. post- 71.08, *p* = 0.020), with significant gains in “Helping Role” (*p* = 0.005), “Teaching-Coaching” (*p* < 0.001), and “Ensuring Quality” dimensions (*p* < 0.001). Correlations between SDL skills and competencies perceived were weaker in the postpandemic group.

**Conclusions:** The perceived competencies have improved while the SDL skills slightly declined in the postpandemic group, suggesting new needs of graduates in the transition to their professional role. The weak correlation between SDL skills and competencies in the postpandemic group underlines the importance of clinical experience in promoting self-direct learning.

**Implications for Nursing Management:** Nurse Managers are required to develop tailored strategies to support the transition process beyond the development of clinical competencies, with greater support for independence in learning—a crucial skill to become resilient and adaptable and to continually face the complexities of modern healthcare.

## 1. Introduction

Nursing education aims to prepare professionals capable of facing the increasing complexity in patient care and the scientific and technological developments in healthcare settings [1]. At the point of their graduation, Nurse Managers and the whole health system [2] expect to have competent nurses capable of addressing their needs and learning continuously, thus equipped with self-directed learning (SDL) skills [3]. However, while competence possessed at the time of graduation has been extensively measured over the years (e.g., [4]), less attention has been devoted to SDL skills, which are crucial to ensure the readiness to face both the transition to the expected role and the future challenging times. In addition, few data have been provided on changes in the competencies as perceived by newly graduated nurses at the time of graduation in the pre- and postpandemic times [5, 6]. Moreover, to our best knowledge, no data has been documented regarding the SDL skills as possessed before and after the pandemic. Beyond the expected competencies, the pandemic has raised awareness of the importance of SDL, not only as a response to crisis situations, but also as a key component of continuing professional development [7]. Nurse Managers responsible for shaping the transition of graduates into nurses should be supported with up-to-date knowledge about the profile of graduates both in SDL skills and competencies possessed who will be trained in the coming years after the pandemic. Therefore, the main intention of this study was to fill the gaps in literature.

## 2. Background

SDL, defined as a process in which the individual takes the initiative in diagnosing his or her own learning needs, formulating objectives, identifying resources, implementing strategies and evaluating results, is particularly relevant in a context where continuous changes are increasing [8]. Possessing SDL skills may facilitate the transition from being a student to a novice nurse [8], to cover the gaps left by education and to face the increased demands of healthcare settings.

Prior to the pandemic, the transition of newly graduated nurses took place in a relatively stable environment, where wards were generally structured to provide adequate support [9] and equipped with preceptorship resources [10]. The culture and routines of the units were also essentially stable; differently, several changes occurred during the pandemic leading to a so-called routine disruption (e.g., [11]), which also brought new challenges for new graduates. The coronavirus disease-2019 (COVID-19) pandemic has radically transformed the healthcare facilities and global education systems, forcing a rapid transition to distance learning and challenging traditional teaching methods [12]. Institutions and educators have had to quickly adapt to distance learning modes, including online platforms and blended learning approaches [13], which triggers several issues [14] on expected competencies, that of SDL skills included, resulting in increasing concerns regarding the effectiveness of education compared to traditional approaches [15].

In this context of uncertainty and rapid change, SDL skills have become even more crucial as students have been forced to take greater responsibility for their own learning and adapt to new models of education [16]. Educators and students have had to adapt quickly to online platforms and blended learning approaches, facing challenges in communication, increased anxiety and concerns about the effectiveness of online learning [17, 18]. However, this shift to distance learning has also provided opportunities to develop independent learning, critical thinking and problem-solving skills; indeed, with the increased reliance on online resources and self-guided study, students have had the opportunity to explore different sources of information and develop a deeper understanding of the subject matter [19]. The flexibility of distance learning allowed students to take control of their own learning process and customize their studies according to their individual preferences and pace [20].

Alongside the SDL skills, the level of professional competence as perceived by recently graduated nurses is also an issue. Due to rationed clinical rotations and limited access to some departments, nursing students had fewer clinical opportunities (e.g., in pressure injury care [21]). Overall, perceived competencies at the time of graduation were lower in the first post-COVID-19 generation (graduated in November 2020) compared to those who graduated just before the pandemic (in November 2019) and higher in subsequent cohorts (November 2021 and 2022) [5]. In addition, a positive correlation between nursing students' SDL skills and their perceived level of competence at the time of graduation was documented in the prepandemic period [22]. This correlation suggests that SDL plays an important role in professional development and maintaining competence in complex healthcare situations. However, no data are available on whether and to what extent there is a correlation between the competencies and SDL skills perceived by newly graduated nurses in the postpandemic period compared to the previous period: the extent to which perceived competencies may be influenced by existing SDL skills, as learning needs may trigger the search for clinical opportunities to acquire competencies in need of development, suggesting that the relationship between these two constructs should be explored. Considering that the COVID-19 pandemic has significantly hindered traditional educational methods, this study aims to examine perceived SDL skills and competencies at the time of graduation compared to those perceived before the pandemic; the secondary aim was to assess correlations between SDL skills and competencies before and after the pandemic.

## 3. Methods

### 3.1. Study Design

A repeated cross-sectional study was conducted. This design is also referred to as a “pseudo longitudinal” study, as the same information is requested in each wave of an independent sample and the analysis is conducted at an aggregated level. This study design was chosen because graduates change over the years and are considered as a cohort exposed to similar educational experiences [23]. Two cross-sectional studies were therefore conducted, one before and one after the pandemic; the whole research project was designed and here reported according to the Strengthening of the Reporting of Observational Studies in Epidemiology checklist [24] (STROBE, see Supplementary Table 1).

### 3.2. Setting and Sample

There were approached two universities, located in the North of Italy with homogeneous nursing programs and extensive network in research since 2016 [25]. There were eligible newly graduates in November 2019 (hereinafter, prepandemic) and in November 2023 (postpandemic group), willing to participate in the study.

### 3.3. Data Collection Process and Tool

The same data collection procedures and instruments [26] were used at the end of the 2018/2019 academic year and at the end of the 2022/2023 academic year. Newly graduated nurses were approached and, after having informed them about the aims of the study and its procedures, an online link was sent out and remained open for approximately 1 month. The EUSurvey website (https://ec.europa.eu/eusurvey/home/welcome) served as the data collection platform. Participation was encouraged through emails, text messages or phone calls.

Overall, the data collection tool was composed of three sections:

- Personal data: In addition to some demographic information (e.g., age, gender) and previous experiences (study and work), the postpandemic group was also asked to report on the academic activities they had attended at the time of the outbreak and the clinical rotations they had completed from the beginning of the COVID-19 pandemic until graduation (e.g., number of weeks, settings, restrictions on access to departments due to the pandemic). They were asked to report on the preceptorship model experienced during clinical rotations and the perceived [1] safety (from not at all to very much) [2], preparedness, and [3] satisfaction with the nursing program's management of the pandemic (from very low to very high). Interruptions to clinical rotations due to quarantine and contagion were also explored, if any.
- The Self-Rating Scale of SDL (SRSSDL) [27]: there was used the Italian validated version (SRSSDL_ITA_) reporting good psychometric properties [28]. The scale provides 60 items categorized in five factors as following: (1) “Awareness” (12 items) addressing learners' understanding of the factors contributing to SDL; (2) “Learning Strategies” (12 items) outlining various strategies that self-directed learners should adopt to develop their skills; (3) “Learning Activities” (12 items) specifying the necessary activities learners should engage in to become self-directed; (4) “Evaluation” (12 items) identifying specific characteristics that help learners monitor their learning progress; and (5) “Interpersonal Skills” (12 items) relating to learners' abilities in interpersonal relationships, which are essential for becoming self-directed learners [28]. Each item – and therefore each factor - was rated on a five-point Likert scale as follows: 1 (never), 2 (seldom), 3 (sometimes), 4 (often) and 5 (always).
- The Nurse Competence Scale (NCS) [29] extensively validated [4, 26] also in the Italian context [30, 31]. The NCS is structured into two parts, the first of which consists of 73 items grouped into seven dimensions: (1) “Helping role” (7 items) as the competence to establish a therapeutic connection with patients through support, presence, empowerment, and guidance; (2) “Teaching-coaching” (16 items), as the competence to facilitate the learning and the understanding of illness and healing in everyday life; (3) “Diagnostic function” (7 items) as assessing, identifying conditions, predicting issues, and recognizing recovery potentialities; (4) “Managing situations” (8 items), as identifying and responding to worsening situations; (5) “Therapeutic intervention” (10 items), including actions to monitor and prevent further health complications; (6) “Ensuring quality” (6 items) involving risk prevention, ensuring safety, and integrating evidence-based practices; and (7) the “Working role” (19 items) including setting priorities and collaborating within the team. Newly graduates were asked to rate their perceived competence using a visual analogue scale (VAS) from 0 (low competence) to 100 (very good competence); scores below 25 indicated “Low level of competence,” between > 25 and 50 “Rather good competence,” between > 50 and 75 “Good competence,” while scores above 75 resulted in a “Very good level of competence” [4, 26]. In the second part, they were asked to indicate how frequently they used each competence during their clinical rotations (on a Likert scale from 0 “Not Applicable”, 1 “Very seldom”, 2 “Occasionally” to 3 “Very often”). In our sample, the NCS demonstrated strong internal consistency with a Cronbach's *α* of over 0.90 across all groups and in both parts of the NCS.

The data collection instrument was piloted in both surveys with newly graduated nurses who were not eligible for the survey to assess its feasibility and comprehensibility. No changes were required.

### 3.4. Ethical Issues

The research protocol was approved by the Internal Review Board of Udine University, Italy (Number 232/2023). New graduates were informed about the aims of the study, the voluntary nature of participation, and the confidentiality of the data. Then, the informed consent was provided. Permissions to use the NCS were granted in the initial study involving Italian researchers [26] from the developer of the scale (Prof. Riitta Meretoja). Likewise, permission to use the Italian version of the SRSSDL_ITA_ was obtained from the author (Dr. Lucia Cadorin). The collected data was securely stored in databases accessible only to researchers and the data collected was analyzed in an aggregate manner.

### 3.5. Rigor

To ensure consistency, the same validated data collection instruments were adopted over the surveys; moreover, new graduates were recruited from universities offering homogenous education pathways and under the same rules before, during and after the pandemic, thus preventing variations in the competencies and SDL skills achieved. Furthermore, the study protocol was the same to ensure consistency in the research process and the STROBE checklist [24] was strictly followed.

### 3.6. Data Analysis

Descriptive statistics were used to summarize the demographic and nursing education characteristics of the participants. Continuous variables, such as age, SRSSDL_ITA_ and NCS scores were expressed in means and 95% confidence interval (CI), while categorical variables were presented as frequencies and percentages. To compare differences between the pre- and the postpandemic demographic, SRSSDL_ITA_ and NCS data, Chi-square and *t*-Test was used. The correlation between the factors/dimensions of the scales was also assessed in both pre- and postpandemic groups using Pearson's correlation that was considered weak if < 0.30, moderate if < 0.70, and strong if > 0.71 [32]. The *p* value significances were set at < 0.05.

## 4. Results

### 4.1. Participants

A total of 336 (participation rate: 97.1%) and 211 (90.1%) graduates took part as the prepandemic and postpandemic groups, respectively. Their average age was significantly higher in the postpandemic group (24.8 years, CI 95% 24.15–25.51) than in the prepandemic group (23.6 years, CI 95% 23.2–23.9), (*p* < 0.001), while the number of women was slightly nonsignificantly lower in the postpandemic group (78.9%) compared to the prepandemic group (81.8%), (*p* = 0.47).

Most graduates in the postpandemic group lived with their families (81.5%) and a small proportion (6.6%) reported having children. The majority attended high school (59.7%) and reported an average final grade of 79.3 out of 100. There was less previous university experience in the postpandemic group (73.5) than in the prepandemic group (88.7%), (*p* < 0.001). There was also a significant difference in previous work experience: 54% in the postpandemic compared to 33.9% in the prepandemic group (*p* < 0.001) (Table 1).

### 4.2. Clinical Education From the Beginning of the COVID-19 Pandemic to Graduation

Table 2 provides a summary of the clinical experiences of the postpandemic group from the onset of the pandemic (March 2020) up to graduation (November 2023). Overall, 65.9% of participants reported that they were attending lessons during the COVID-19 onset. They had attended an average of 0.2 (CI 95% 0.1–0.3) placements weeks before the pandemic and 72.9 (CI 95% 47.8–98.1) weeks after the outbreak, of which 1.3 (CI 95% 0.9–1.6) consisted of distance learning. In total, less than half were exposed to COVID + patients, also because they were forbidden to access units caring for infected patients (44.1%).

Most participants (85.8%) were supervised by a clinical nurse. They perceived themselves to be prepared to face clinical rotations (from high 61.2% to very highly 37.9%) as well as they perceived being safe during the clinical rotations (from somewhat 58.8% to a great extent 38.4%). The clinical learning was interrupted by around one out of three participants at least once due to COVID + cases among patients or healthcare workers (17.1%) and due to contact with COVID + cases in their personal life (12.8%). Half of the participants reported being infected mainly at home (46.4%) whereas 12.8% contracted COVID-19 during clinical placements.

Participants expressed high (66.8%) and very high (26.6%) levels of satisfaction with the Nursing Program management of the COVID-19 outbreak; only a small proportion expressed low and very low satisfaction (5.7% and 0.9%, respectively).

### 4.3. Perceived SDL Skills

At the SRSSDL_ITA_ (Table 3) significant differences emerged when comparing factors' scores between the pre- and the postpandemic group. Overall, the total score decreased (from 4.27 out of 5 to 4.18, *p* = 0.030). Except for “Attitudes” (pre- 4.33 vs. postpandemic 4.36, *p* = 0.502), “Motivation” (pre- 4.29 vs. postpandemic 4.29, *p* = 0.902) and “Learning activities” (pre- 4.25 vs. postpandemic 4.16, *p* = 0.120), there were significant changes in the other factors. Four factors deteriorated significantly from the prepandemic to the postpandemic group, namely “Awareness” (4.41 vs. 4.28, *p* = 0.005), “Learning Strategies” (4.38 vs. 3.86, *p* ≤ 0.001), “Interpersonal skills” (4.29 vs. 4.12, *p* = 0.007) and “Constructing knowledge” (3.81 vs. 3.60, *p* = 0.030). Differently, as for “Learning methods”, the score in the postpandemic (4.30) was significantly higher than in the prepandemic group (3.99) (*p* ≤ 0.001).

### 4.4. Perceived Competencies and Frequency of Use

As shown in Table 4, the perceived overall competencies were higher in the postpandemic than in the prepandemic group (71.08 out of 100; CI 95%, 68.97–73.20 vs. 68.01 CI 95% 66.49–69.53, *p* = 0.020). There were no differences in the dimensions “Diagnostic functions”, “Managing situations” and “Therapeutic interventions”. Differently, in the dimensions “Helping role” (72.16 vs. 75.80, *p* = 0.005), “Teaching-coaching” (67.12 vs. 72.18, *p* ≤ 0.001), “Ensuring quality” (63.75 vs. 70.44, *p* < 0.001) and “Work role” (68.07 vs. 71.27, *p* = 0.030), the postpandemic group reported significantly higher competencies than the prepandemic group. Overall, the frequency of use of these competencies was homogeneous between the groups (*p* = 0.246); however, there was a slight decrease with a significant decrease in the postpandemic group compared to the prepandemic group in the dimensions “Helping role” (2.47 vs. 2.37, *p* = 0.004) and “Managing the situation” (2.38 vs. 2.31, *p* = 0.012).

### 4.5. Correlations Between Perceived SDL Skills and Competencies

In the prepandemic group, there was a moderate correlation between the overall SRSSDL_ITA_ score and the NCS score (*ρ* = 0.433, *p* < 0.001), while the correlations between the factors of each tool ranged from 0.113 to 0.386, with most being statistically significant (*p* < 0.001) (Supplementary Table 2). Among the postpandemic group, there was a moderate correlation between the SRSSDL_ITA_ total score and the NCS score (*ρ* = 0.343, *p* < 0.001), while correlations between factors ranged from 0.098 to 0.356, with the fewest being statistically significant (Supplementary Table 3).

## 5. Discussion

To our knowledge, this is the first study to compare the perceptions of recently graduated nurses who completed their education entirely after COVID-19 came into effect—from the academic year 2020/21 to 2022/23—with those of nurses who completed their education immediately before it came into effect in 2019. A 5-year time span does not reflect a true generation, which typically requires 15/20 years. However, since the participants experienced two different historical moments that impacted their education [33], we can consider the groups involved in our study as two different generations of nurses. Comparing their perceived SDL skills and competencies at the time of graduation may inform the proper development of strategies to effectively manage their transition into practice and subsequent professional development.

### 5.1. Participants and Clinical Education From the Beginning of Pandemic to Graduation

Overall, while the main demographic characteristics were in line with those documented before the pandemic [26, 34], the new generation seems to decide to undertake the nursing degree more than 1 year later as a second choice both after work and/or university experiences. On the one hand, this may enrich the nursing profession and teams with graduated people possessing diverse experiences; however, on the other, this delay may suggest that the young do not have a clear picture of the nursing role, thus requiring additional time to understand its potential. Overall, this delay may also express the effect of the pandemic, during which nurses became heroes and their role more attractive also toward those who were already pursuing other professional or academic careers. The persistence of this new profile of graduates should be monitored in the future, given that it may also affect the transition process in the working role.

At the COVID-19 onset, most participants were attending lessons; overall, the number of weeks spent on distance clinical learning surrogating the direct experience in the units, according to the restrictions imposed by the hospitals, was limited as compared to previous cohorts [5]. Students were supervised mainly by clinical nurses appointed for this role, and they perceived to be prepared and safe when facing the units, recognizing the role of their nursing programs in well-managing the challenging times. Almost half of them were barred from accessing COVID-19 units, preventing direct care for these patients, suggesting that the training and hospital system is cautiously introducing students to the real world. Overall, most of their clinical training took place outside the COVID-19+ units, in the remaining so-called ‘clean' facilities, where they experienced interruptions due to the spread of infection despite attempts to protect them. Overall, the newly post-COVID-19 graduates acquired their clinical competencies in protected environments; however, changes in the learning pathway from their enrollment in nursing education (e.g., stopping and re-starting the clinical rotations due to interruptions), as well as the challenges lived by the units where they undertook their rotations (e.g., due to the different protocols delivered by healthcare settings during the pandemic), may have stimulated their SDL skills.

### 5.2. Perceived SDL Skills

The overall perceived SDL skills has slightly declined—although the practical meaning of the difference (from 4.27 to 4.18) is limited. As compared to traditional nursing education, and despite the increased emphasis on independent learning brought on by the pandemic, our students at the point of graduation perceived less SDL skills as before. Overall, Italian students still have high SDL skills compared to other countries [35], while distance learning, where students were expected to take greater responsibility for their learning processes [36], had limited impact. This can also be interpreted as a ceiling effect, as the students were already well skilled and therefore the improvements are limited; however, it can also be interpreted as a biased finding, where graduates have overestimated their skills, both before and after the pandemic (or only in one group).

Apart from attitudes and motivations, which remained stable, thus suggesting similar skills in identifying the learning needs and in the degree of motivation to learn, two main trends have emerged: most of the SRSSDL_ITA_ factors significantly – although slightly - declined in their scores, suggesting that graduates perceived themselves less equipped with the capacity to plan, conduct and self-evaluate the learning process. Also in this case, the differences have little practical implications, apart from that regarding the learning strategies (from 4.38 to 3.86), where the decline is more evident.

During the pandemic, students may have been exposed to limited support and tutorial sessions delivered by nurse educators who aimed at coaching independence in learning; on the other hand, the massive digitalization may have prevented the development of self-learning skills, given the ample amount of material provided (e.g., video recording of the lessons). However, in the learning methods newly graduates have significantly increased their skills (from 3.99 to 4.30), possibly because of the rapid shift to digital learning environments, which may have engaged students more actively with online platforms, self-paced materials, and various digital tools, all tools to be independently navigated [37]. Furthermore, the autonomy in accessing educational content and managing learning tasks without direct supervision may have contributed to a heightened sense of competence in the learning methods [38]. Thus, while other areas of SDL, such as planning and self-evaluation, showed a decline, the need to adapt to a digitalized learning environment may have resulted in graduates feeling more confident about their capacity to utilize diverse learning methods. This seems to suggest that the shift toward digital education, despite being challenging, provided students with opportunities to enhance certain aspects of their SDL skills, particularly in adapting to and mastering digital learning techniques.

### 5.3. Perceived Competencies and Frequency of Use

Postpandemic graduates perceived higher competencies, suggesting that their clinical learning experiences were effective in preparing them for the expected role as compared to the prepandemic group. They considered themselves almost ready to start the practice, given that overall scores are all above 71 out of 100, indicating sufficient competence [29]. The increased scores suggested, in order, more competence in “Ensuring quality” (7 points gained out of 100) indicating that the strategies used to ensure safety and prevent risk during the pandemic affected the competencies acquired; then, participants reported more competencies in teaching and coaching patients, in the helping and working role, suggesting that they have been better coached in safety issues and soft skills. In contrast, some traditional competencies (“Diagnostic Functions”, “Managing Situations”, “Therapeutic Interventions”) remained stable across the groups. While the high perceived competence in quality of care may be related to the efforts of health services in teaching and promoting safety, other findings can be interpreted from other perspectives: Students may have enhanced their soft skills in an attempt to limit the communication barriers imposed by personal protective equipment (e.g., wearing masks). Moreover, the pandemic itself helped nurses expand their role capacities and provide education (e.g., how to prevent the spread of the infection), so students may have been exposed to positive role models. Notably, despite the decrease in the frequency of use in several competencies, as already underlined in other fields [21], students have gained the expected competencies, suggesting that they may have appreciated each learning moment, maximizing its effectiveness.

In the context of literature available, the competencies perceived before the pandemic were overall and, in all dimensions, slightly higher than those reported at the European level in those times [26]. In the postpandemic a notable improvement has emerged in some dimensions (e.g., “Helping Role”, “Ensuring Quality”), reflecting the heightened focus on patient care and safety protocols during the pandemic. However, in the “Therapeutic intervention”, a decline has emerged, likely because of the complexities in the treatment and the reduced frequency of competency use, in addition to the limitations in clinical placements and increased reliance on virtual learning [26]. Overall, the perceived high level of competence indicates a form of empowerment of graduates [39] that has been documented as increasing the intention to remain in the profession [22, 40].

### 5.4. Correlations Between Perceived SDL Skills and Competencies

Overall, weak positive correlations emerged between SRSSDL_ITA_ and NCS, around 0.300 in the prepandemic group and falling even lower in the postpandemic group. Notably, several correlations in the postpandemic group did not reach statistical significance, highlighting a shift in the relationship between SRSSDL_ITA_ and NCS.

SDL skills are fundamental in fostering professional growth and readiness for clinical roles and may cultivate autonomy and confidence—which have been underlined as critical traits for graduates transitioning into clinical practice [41]. However, weak correlations suggest that SDL skills and professional competencies have limited relation [42], especially in the post pandemic times. The clinical learning environment is a mediator between SDL and competencies. Mentorship and real-world experiences are essential in translating SDL into effective practice [43]. Therefore, the decline in significant correlations in the postpandemic group may be seen as an effect of the challenges posed by the untraditional clinical learning exposure and altered unit experiences. Without adequate real-world practice, SDL may not fully translate into the practical competencies required in nursing roles. Studies conducted in more stable educational settings have reported moderate correlations between SDL and competencies [35], confirming the importance of clinical experience in reinforcing this relationship. Therefore, on all occasions in the future where clinical practice experience is limited, nursing programs should compensate for this deficiency with appropriate strategies and enhanced mentorship programs to ensure that SDL skills are effectively coached and nurtured.

### 5.5. Limitations

This study has several limitations. First, a convenience sample of graduates has been involved, and this may affect the generalization of the findings. Secondly, data were collected at the time of graduation: studies suggest that graduates' perceived competencies decrease after they enter the profession [44] due to challenges in daily practice that may cause uncertainty. Therefore, longitudinal studies are recommended to also assess self-perceived competence over time. Third, only self-assessments were used, so the results may lead to an over- or underestimation of competencies: external assessments (e.g., by preceptors) are recommended to increase the robustness of the results.

### 5.6. Nurse Manager Implications

SDL skills appear to have decreased slightly, while general skills have improved slightly. As a result, Nurse Managers should pay less attention to improving the competencies of the newly hired, while SDL skills require greater investment. The latter are usually expected as a result of academic preparation, while practical competencies are often seen as an indicator of a successful clinical learning rotation. Although these findings should be considered by educators, it can be challenging for nurse managers to focus on soft skills—such as SDL skills—rather than practical skills due to time constraints, system efficiencies and the need to provide direct care. However, even if these findings are confirmed, Nurse Managers should support the development of SDL skills by facilitating the expression of learning needs, setting goals and establishing appropriate strategies and methods for continuous learning. In the transition phase, this support should be more targeted and consider the need for individualized strategies for induction. In contexts where it is not possible to use dedicated preceptors due to a lack of human resources, compensatory strategies developed by the Nurse Manager—such as guidance on creating a personal portfolio, frequent meetings and constructive feedback—should be used.

## 6. Conclusions

This investigation provides findings in a context where few studies have examined how the pandemic has impacted nursing education outcomes. This is of critical importance to Nurse Managers who have a responsibility to develop transition strategies for new graduates.

Overall, the first new post COVID-19 graduates are ready for practice, more than the past: they self-reported overall competencies exceeding those gained in prepandemic times, especially in patient safety and quality assurance. This suggests that nursing education has successfully adapted to the challenging time of the pandemic: however, while competencies have improved, the perceived SDL skills have slightly declined. Therefore, Nurse Managers should expect graduates who are ready for practice in the core competencies and are less qualified to take ownership of their learning and thus to develop their professional progress after graduation.

In the transition process, priority setting should go beyond the development of clinical competencies, as there should be greater support for promoting graduate autonomy in learning, a recognized core skill for professional nurses. Nurse Managers should foster an environment and strategies that encourage the development of SDL skills—while simulation-based and peer-learning sessions can help graduates close some gaps, particularly in practical competencies such as therapeutic interventions and diagnostic functions. By encouraging autonomy in learning, Nurse Managers ensure that new nurses are not only competent, but also resilient and adaptable, and prepared to continually meet the changing demands of complex healthcare settings. Further studies are needed to validate the findings and better understand the impact of SDL skills on competency development: in this context, promoting objective measures of competencies and SDL skills, broader samples and longitudinal studies is recommended.

## Figures and Tables

**Table 1:** Participants' main characteristics.

Individual variables, graduate in	Prepandemic group *n* = 336	Postpandemic group *n* = 211	*p* value
Age (years), mean (CI)	23.6 (23.2–23.9)	24.8 (24.15–25.51)	< 0.001
Gender, female, *n* (%)	275 (81.8)	165 (78.9)	0.470
Living with, *n* (%)		172 (81.5)	
With my family	—	22 (10.4)	
With my boy/girlfriend	—	6 (2.9)	
Alone	—	11 (5.2)	
Students/colleagues	—	0 (0.0)	
Other			
With children, *n* (%)	—	14 (6.6)	
Secondary education, *n* (%)	—	126 (59.7)	
High school	—	55 (26.1)	
Technical school	—	29 (13.7)	
Professional school	—	1 (0.5)	
Foreign school			
Secondary school	—	79.3 (77.6–80.8)	
Grade (score 60–100), mean (CI)			
Previous university experience, *n* (%)	298 (88.7)	155 (73.5)	< 0.001
Previous work experience, *n* (%)	114 (33.9)	114 (54.0)	< 0.001

*Note: n*, number.

Abbreviation: CI, confidence interval.

**Table 2:** Postpandemic group (*n* = 211): clinical experiences from the COVID-19 onset up to graduation.

	Postpandemic group *n* = 211
Academic activities followed at the COVID-19 outbreak onset, *n* (%)	
I was attending clinical placements	5 (2.4)
I was following lessons	139 (65.9)
I was attending examination(s)	5 (2.4)
I was abroad (Erasmus experience)	62 (29.3)
I was following laboratories	0 (0.0)
Other	0 (0.0)
Clinical placements attended, number, mean (CI) up to the COVID-19 outbreak onset	0.2 (0.1–0.3)
Learning experiences attended after the COVID-19 outbreak onset	
At the ward level, weeks	72.9 (47.8–98.1)
Distance learning, weeks	1.3 (0.9–1.6)
Restrictions to access some units with COVID + patients, *n* (%)	93 (44.1)
Preceptorship model, *n* (%) I was supervised by	
A clinical nurse	181 (85.8)
The nursing staff	6 (2.8)
The head nurse and a clinical nurse	1 (0.5)
The nurse teacher	0 (0.0)
The head nurse	0 (0.0)
A clinical nurse and the nurse teacher	23 (10.9)
Perceived preparedness to deal with the clinical rotation, *n* (%)	
Very low	0 (0.0)
Low	2 (0.9)
High	129 (61.2)
Very high	80 (37.9)
Perceived safety, *n* (%)	
Not at all	0 (0.0)
Very little	6 (2.8)
Somewhat	124 (58.8)
To a great extent	81 (38.4)
Interruptions for quarantine	
Yes, for COVID + cases among patients'/healthcare workers, *n* (%)	36 (17.1)
Yes, for COVID + cases among out of hospital contacts, *n* (%)	27 (12.8)
Contagion, *n* (%)	
Yes, during my clinical placements	27 (12.8)
Yes, at home	98 (46.4)
I don't know	13 (6.2)
No, never	73 (34.6)
Degree of satisfaction regarding the COVID-19 outbreak nursing program management	
Very low	2 (0.9)
Low	12 (5.7)
High	141 (66.8)
Very high	56 (26.6)

*Note: n*, number.

Abbreviations: CI, confidence interval; COVID-19, coronavirus disease-2019.

**Table 3:** Self-Rating Scale for Self-Directed Learning (SRSSDL_ITA_) scores in the pre- and in the postpandemic graduated nurses.

Factors, score^∗^, mean (CI)	Prepandemic group *n* = 336	Postpandemic group *n* = 211	*p* value
Awareness	4.41 (4.30–4.46)	4.28 (4.20–4.36)	0.005
Attitudes	4.33 (4.283–4.38)	4.36 (4.29–4.43)	0.502
Motivation	4.29 (4.23–4.35)	4.29 (4.20–4.37)	0.902
Learning strategies	4.38 (4.32–4.43)	3.86 (3.76–3.97)	< 0.001
Learning methods	3.99 (3.92–4.07)	4.30 (4.22–4.38)	< 0.001
Learning activities	4.25 (4.19–4.32)	4.16 (4.06–4.26)	0.120
Interpersonal skills	4.29 (4.23–4.35)	4.12 (4.02–4.23)	0.007
Constructing knowledge	3.81 (3.70–3.91)	3.60 (3.44–3.76)	0.030
Overall	4.27 (4.23–4.32)	4.18 (4.11–4.25)	0.030

*Note: n*, number.

Abbreviations: CI, confidence interval; COVID-19, coronavirus disease-2019.

^∗^The Self-Rating Scale for Self-Directed Learning (SRSSDL_ITA_), at the competency level, is measured by a Likert scale from never (=1) to always (=5).

**Table 4:** Nurse Competence Scale (NCS) scores in the pre- and in the postpandemic graduated nurses.

Dimension, score^∗^, mean (CI)	Prepandemic group *n* = 336	Postpandemic group *n* = 211	*p* value
Helping role	72.16 (70.66–73.66)	75.8 (73.78–77.83)	0.005
Frequency of using competency^∗∗^	2.47 (2.43–2.51)	2.37 (2.31–2.42)	0.004
Teaching-coaching	67.12 (65.4–68.84)	72.18 (70.01–74.35)	< 0.001
Frequency of using competency	2.24 (2.19–2.28)	2.21 (2.14–2.27)	0.459
Diagnostic functions	70.17 (68.43–71.9)	72.49 (70.33–74.65)	0.099
Frequency of using competency	2.38 (2.34–2.43)	2.24 (2.18–2.31)	0.079
Managing situation	70.24 (68.47–72.01)	71.05 (68.83–73.28)	0.574
Frequency of using competency	2.38 (2.34–2.42)	2.31 (2.25–2.37)	0.012
Therapeutic interventions	65.96 (64.16–67.75)	65.09 (62.47–67.7)	0.590
Frequency of using competency	2.32 (2.27–2.36)	2.17 (2.10–2.23)	0.148
Ensuring quality	63.75 (61.76–65.74)	70.44 (67.96–72.92)	< 0.001
Frequency of using competency	2.12 (2.07–2.17)	2.14 (2.07–2.21)	0.724
Work role	68.07 (66.28–69.86)	71.27 (68.99–73.56)	0.030
Frequency of using competency	2.27 (2.23–2.31)	2.23 (2.17–2.29)	0.237
Overall competencies	68.01 (66.49–69.53)	71.08 (68.97–73.20)	0.020
Frequency of using competencies	2.30 (2.26–2.33)	2.23 (2.18–2.29)	0.246

*Note: n*, number.

Abbreviations: CI, confidence interval; COVID-19, coronavirus disease-2019.

^∗^The NCS, at the competency level, is measured by a visual analogue scale, where 0 indicates a very low level and 100 indicates a high level of competency.

^∗∗^The frequency of using the competences, from ‘very seldom' (= 1) to ‘occasionally' (= 2) and to ‘very often' (= 3).

## Data Availability

Data are available at the request from the authors.

## Supporting Information

Supporting Table 1. Strengthening the Reporting of Observational studies in Epidemiology (STROBE) Statement: cross-sectional studies [24].

Supporting Table 2. Correlations between the Nurse Competence Scale (NCS) [29] score and the Self-Rating Scale of Self-Directed Learning (SRSSDL_ITA_) [28] scores: prepandemic group.

Supporting Table 3. Correlations between the Nurse Competence Scale (NCS) [29] score and the Self-Rating Scale of Self-Directed Learning (SRSSDL_ITA_) [28] scores: postpandemic group.

## References

[1] Fawaz M., Hamdan-Mansour A. M., Tassi A. (2018). Challenges Facing Nursing Education in the Advanced Healthcare Environment. *International Journal of Africa Nursing Sciences*.

[2] Purabdollah M., Zamanzadeh V., Ghahramanian A., Valizadeh L., Mousavi S., Ghasempour M. (2023). Competencies Expected of Undergraduate Nursing Students: A Scoping Review. *Nursing Open*.

[3] Grealish L., van de Mortel T., Brown C. (2018). Redesigning Clinical Education for Nursing Students and Newly Qualified Nurses: A Quality Improvement Study. *Nurse Education in Practice*.

[4] Flinkman M., Leino-Kilpi H., Numminen O., Jeon Y., Kuokkanen L., Meretoja R. (2017). Nurse Competence Scale: A Systematic and Psychometric Review. *Journal of Advanced Nursing*.

[5] Dentice S., Chiappinotto S., Kajander-Unkuri S., Grassetti L., Brugnolli A., Palese A. (2024). Perceived Competences by Graduated Nurses Before and During COVID-19 Restrictions: A Repeated Cross-Sectional Study From 2019 to 2022. *Nurse Education in Practice*.

[6] McGarity T., Monahan L., Acker K., Pollock W. (2023). Nursing Graduates’ Preparedness for Practice: Substantiating the Call for Competency-Evaluated Nursing Education. *Behavioral Sciences*.

[7] Berdida D. J. E. (2023). Resilience and Academic Motivation’s Mediation Effects in Nursing Students’ Academic Stress and Self-Directed Learning: A Multicenter Cross-Sectional Study. *Nurse Education in Practice*.

[8] Cadorin L., Suter N., Dante A., Williamson S. N., Devetti A., Palese A. (2012). Self-Directed Learning Competence Assessment Within Different Healthcare Professionals and Amongst Students in Italy. *Nurse Education in Practice*.

[9] Doughty L., McKillop A., Dixon R., Sinnema C. (2018). Educating New Graduate Nurses in Their First Year of Practice: The Perspective and Experiences of the New Graduate Nurses and the Director of Nursing. *Nurse Education in Practice*.

[10] van Rooyen D. R. M., Jordan P. J., Ten Ham-Baloyi W., Caka E. M. (2018). A Comprehensive Literature Review of Guidelines Facilitating the Transition of Newly Graduated Nurses to Professional Nurses. *Nurse Education in Practice*.

[11] Gabay G., Tarabeih M. (2021). Underground COVID-19 Home Hospitals for Haredim: Non-Compliance or a Culturally Adapted Alternative to Public Hospitalization?. *Journal of Religion and Health*.

[12] Dewart G., Corcoran L., Thirsk L., Petrovic K. (2020). Nursing Education in a Pandemic: Academic Challenges in Response to COVID-19. *Nurse Education Today*.

[13] Seymour-Walsh A. E., Bell A., Weber A., Smith T. (2020). Adapting to a New Reality: COVID-19 Coronavirus and Online Education in the Health Professions. *Rural and Remote Health*.

[14] Hunck S., Engelhard K., Mildenberger P., Kurz S. (2022). Chances and Challenges of Increasing Digitalization of Teaching in the Discipline Anesthesiology From the Perspective of Students. *Anaesthesiologie*.

[15] Ameri H., Mahami-Oskouei M., Sharafi S., Saadatjoo S., Miri M., Arab-Zozani M. (2023). Investigating the Strengths and Weaknesses of Online Education During COVID-19 Pandemic From the Perspective of Professors and Students of Medical Universities and Proposing Solutions: A Qualitative Study. *Biochemistry and Molecular Biology Education*.

[16] Lee S., Kim D. H., Chae S. M. (2020). Self-Directed Learning and Professional Values of Nursing Students. *Nurse Education in Practice*.

[17] Carrillo C., Flores M. A. (2020). COVID-19 and Teacher Education: A Literature Review of Online Teaching and Learning Practices. *European Journal of Teacher Education*.

[18] Kartsoni E., Bakalis N., Markakis G., Zografakis-Sfakianakis M., Patelarou E., Patelarou A. (2023). Distance Learning in Nursing Education During the COVID-19 Pandemic: Psychosocial Impact for the Greek Nursing Students-A Qualitative Approach. *Healthcare (Basel)*.

[19] Janes G., Ekpenyong M. S., Mbeah-Bankas H., Serrant L. (2023). An International Exploration of Blended Learning Use in Pre-registration Nursing and Midwifery Education. *Nurse Education in Practice*.

[20] Joo J. Y. (2024). Abrupt Transition to Remote Learning in Nursing Students During the COVID-19 Pandemic. *Journal of Nursing Education*.

[21] Zito M., Chiappinotto S., Galazzi A. (2024). Nursing Students’ Knowledge, Attitudes and Learning Occasions About Pressure Injuries at the Time of Graduation: A Multi-Method Pre-Post Pandemic Study. *Journal of Tissue Viability*.

[22] Visiers-Jiménez L., Palese A., Brugnolli A. (2022). COMPEUnurse-Consortium. Nursing Students’ Self-Directed Learning Abilities and Related Factors at Graduation: A Multi-Country Cross-Sectional Study. *Nurs Open*.

[23] Yee J. L., Niemeier D. (1996). Advantages and Disadvantages: Longitudinal vs. Repeated Cross-Section Surveys. *Project Battelle*.

[24] von Elm E., Altman D. G., Egger M. (2014). The Strengthening the Reporting of Observational Studies in Epidemiology (STROBE) Statement: Guidelines for Reporting Observational Studies. *International Journal of Surgery*.

[25] Palese A., Destrebecq A., Terzoni S. (2016). Validation of the Italian Clinical Learning Environment Instrument (SVIAT): BR Study Protocol. *Assistenza Infermieristica e Ricerca*.

[26] Kajander‐Unkuri S., Koskinen S., Brugnolli A. (2021). The Level of Competence of Graduating Nursing Students in 10 European Countries-Comparison Between Countries. *Nursing Open*.

[27] Williamson S. N. (2007). Development of a Self-Rating Scale of Self-Directed Learning. *Nurse Researcher*.

[28] Cadorin L., Suter N., Saiani L., Naskar Williamson S., Palese A. (2010). Self-Rating Scale of Self-Directed Learning (SRSSDL): Preliminary Results From the Italian Validation Process. *Journal of Research in Nursing*.

[29] Meretoja R., Isoaho H., Leino-Kilpi H. (2004). Nurse Competence Scale: Development and Psychometric Testing. *Journal of Advanced Nursing*.

[30] Dellai M., Mortari L., Meretoja R. (2009). Self-Assessment of Nursing Competencies--Validation of the Finnish NCS Instrument With Italian Nurses. *Scandinavian Journal of Caring Sciences*.

[31] Notarnicola I., Stievano A., De Jesus Barbarosa M. R. (2018). Nurse Competence Scale: Psychometric Assessment in the Italian Context. *Ann Ig*.

[32] Akoglu H. (2018). User’s Guide to Correlation Coefficients. *Turkish Journal of Emergency Medicine*.

[33] Tan S. H. E., Chin G. F. (2023). Generational Effect on Nurses’ Work Values, Engagement, and Satisfaction in an Acute Hospital. *BMC Nursing*.

[34] Stevanin S., Bressan V., Bulfone G., Zanini A., Dante A., Palese A. (2015). Knowledge and Competence With Patient Safety as Perceived by Nursing Students: The Findings of a Cross-Sectional Study. *Nurse Education Today*.

[35] Visiers-Jiménez L., Kuokkanen L., Leino-Kilpi H. (2022). Graduating Nursing Students’ Empowerment and Related Factors: Comparative Study in Six European Countries. *Healthcare (Basel)*.

[36] Alharbi H. F., Alsubaie A., Gharawi R. (2024). The Relationship Between Virtual Simulation, Critical Thinking, and Self-Directed Learning Abilities of Nursing Students in Riyadh, Saudi Arabia. *PeerJ*.

[37] Liu Y., Hu H., Wang L. (2023). Medical Education Environment Perception and Learning Engagement in Undergraduate Nursing Students: The Mediating Effect of Self-Regulated Learning Ability. *Nurse Education in Practice*.

[38] Langegård U., Kiani K., Nielsen S. J., Svensson P. A. (2021). Nursing Students’ Experiences of a Pedagogical Transition From Campus Learning to Distance Learning Using Digital Tools. *BMC Nursing*.

[39] Koskinen S., Brugnolli A., Fuster-Linares P. (2023). A Successful Nursing Education Promotes Newly Graduated Nurses’ Job Satisfaction One Year After Graduation: A Cross-Sectional Multi-Country Study. *BMC Nursing*.

[40] Viottini E., Ferrero A., Acquaro J. (2024). Investire sul Personale Sanitario: Motivi di Iscrizione al Corso di Laurea in Infermieristica. Risultati dello Studio Pilota. *Assistenza Infermieristica e Ricerca*.

[41] Sheppard-Law S., Curtis S., Bancroft J., Smith W., Fernandez R. (2018). Novice Clinical Nurse Educator’s Experience of a Self-Directed Learning, Education and Mentoring Program: A Qualitative Study. *Contemporary Nurse*.

[42] Peng Y., Tan L., Zhang K., Zhu N., Dong H., Gao H. (2024). The Mediating Role of Nutritional Care Literacy on the Relationship Between Self-Directed Learning Ability and Nursing Competence. *BMC Nursing*.

[43] Saiani L. (2024). Si Può Aumentare il Numero di Iscritti ai Corsi per Infermieri?. *Assistenza Infermieristica e Ricerca*.

[44] Mesaglio M., Dentice S., Grassetti L. (2024). The Role of the Simulation in Supporting Newly Graduated Nurses in Their First 5 Months of Working Transition: Findings From a Mixed-Method Study. *Journal of Clinical Nursing*.

