# Supplementary material for: Self-Directed Learning and Competencies as Perceived by New Graduates Before and After the Pandemic: A Repeated Cross-Sectional Study
Source: J Nurs Manag. 2025 May 27;2025:1756024. doi: 10.1155/jonm/1756024 (PMC12133363; doi:10.1155/jonm/1756024)
Supplement: Supporting Information — Additional supporting information can be found online in the Supporting Information section. [file 1756024.f1.docx]

**Supplementary Table 1**. STrengthening the Reporting of OBservational studies in Epidemiology (STROBE) Statement: cross-sectional studies (24)

|  | Item No | Recommendation | Section |
| --- | --- | --- | --- |
| Title and abstract | 1 | (a) Indicate the study’s design with a commonly used term in the title or the abstract | Title, abstract |
|  |  | (b) Provide in the abstract an informative and balanced summary of what was done and what was found | Abstract |
| Introduction | | |  |
| Background/rationale | 2 | Explain the scientific background and rationale for the investigation being reported | Background |
| Objectives | 3 | State specific objectives, including any prespecified hypotheses | Background |
| Methods | | |  |
| Study design | 4 | Present key elements of study design early in the paper | Study design |
| Setting | 5 | Describe the setting, locations, and relevant dates, including periods of recruitment, exposure, follow-up, and data collection | Setting and sample |
| Participants | 6 | (a) Give the eligibility criteria, and the sources and methods of selection of participants | Setting and sample |
| Variables | 7 | Clearly define all outcomes, exposures, predictors, potential confounders, and effect modifiers. Give diagnostic criteria, if applicable | Data collection process and tool |
| Data sources/ measurement | 8 | For each variable of interest, give sources of data and details of methods of assessment (measurement). Describe comparability of assessment methods if there is more than one group | Data collection process and tool |
| Bias | 9 | Describe any efforts to address potential sources of bias | Data collection process and tool |
| Study size | 10 | Explain how the study size was arrived at | Data collection process and tool |
| Quantitative variables | 11 | Explain how quantitative variables were handled in the analyses. If applicable, describe which groupings were chosen and why | Data Analysis |
| Statistical methods | 12 | (a) Describe all statistical methods, including those used to control for confounding | Data Analysis |
|  |  | (b) Describe any methods used to examine subgroups and interactions | Data Analysis |
|  |  | (c) Explain how missing data were addressed | / |
|  |  | (d) If applicable, describe analytical methods taking account of sampling strategy | / |
|  |  | (e) Describe any sensitivity analyses | Data Analysis |
| Results | | |  |
| Participants | 13 | (a) Report numbers of individuals at each stage of study—eg numbers potentially eligible, examined for eligibility, confirmed eligible, included in the study, completing follow-up, and analysed | Results - Participants |
|  |  | (b) Give reasons for non-participation at each stage | / |
|  |  | (c) Consider use of a flow diagram | / |
| Descriptive data | 14 | (a) Give characteristics of study participants (eg demographic, clinical, social) and information on exposures and potential confounders | Results – Participants – Clinical education from the beginning of the COVID-19 pandemic to graduation, Table 1, Table 2 |
|  |  | (b) Indicate number of participants with missing data for each variable of interest | Results |
| Outcome data | 15 | Report numbers of outcome events or summary measures | Results, Clinical education from the beginning of the COVID-19 pandemic to graduation – Perceived competences and frequency of use – New graduated Self-Directed Learning, Table 2, Table 3, Table 4 |
| Main results | 16 | (a) Give unadjusted estimates and, if applicable, confounder-adjusted estimates and their precision (eg, 95% confidence interval). Make clear which confounders were adjusted for and why they were included | / |
|  |  | (b) Report category boundaries when continuous variables were categorized | Results –Perceived competences and frequency of use – Perceived self-directed learning skills, Table 3, Table 4  Supplementary Table 2 and 3 |
|  |  | (c) If relevant, consider translating estimates of relative risk into absolute risk for a meaningful time period | / |
| Other analyses | 17 | Report other analyses done—eg analyses of subgroups and interactions, and sensitivity analyses | Results  Supplementary Tables 2 and 3 |
| Discussion | | |  |
| Key results | 18 | Summarise key results with reference to study objectives | Discussion |
| Limitations | 19 | Discuss limitations of the study, taking into account sources of potential bias or imprecision. Discuss both direction and magnitude of any potential bias | Limitations |
| Interpretation | 20 | Give a cautious overall interpretation of results considering objectives, limitations, multiplicity of analyses, results from similar studies, and other relevant evidence | Discussion |
| Generalisability | 21 | Discuss the generalisability (external validity) of the study results | Conclusions |
| Other information | | |  |
| Funding | 22 | Give the source of funding and the role of the funders for the present study and, if applicable, for the original study on which the present article is based | / |

**Supplementary Table 2**. Correlations between the Nurse Competence Scale (NCS) (29) and the Self Rating Scale of Self-directed Learning (SRSSDL_ITA_) (28) scores: pre-pandemic group

|  |  | **NURSE COMPETENCE SCALE** | | | | | | | |
| --- | --- | --- | --- | --- | --- | --- | --- | --- | --- |
|  | **Correlations** | **Helping Role** | **Teaching-Coaching** | **Diagnostic Functions** | **Managing Situation** | **Therapeutic Intervention** | **Ensuring Quality** | **Working Role** | **Overall Competence** |
| **SELF RATING SCALE OF SELF-DIRECTED LEARNING_ITA_** | **Awareness** | 0.309** | 0.332** | 0.348** | 0.212** | 0.301** | 0.315** | 0.295** | 0.354** |
|  | **Attitutes** | 0.341** | 0.320** | 0.327** | 0.265** | 0.278** | 0.305** | 0.254** | 0.346** |
|  | **Motivation** | 0.276** | 0.312** | 0.335** | 0.227** | 0.304** | 0.284** | 0.237** | 0.329** |
|  | **Learning Strategies** | 0.338** | 0.410** | 0.378** | 0.238** | 0.334** | 0.326** | 0.275** | 0.386** |
|  | **Learning Methods** | 0.290** | 0.309** | 0.299** | 0.200** | 0.270** | 0.227** | 0.270** | 0.319** |
|  | **Learning Activities** | 0.258** | 0.220** | 0.263** | 0.113 | 0.243** | 0.210** | 0.182** | 0.245** |
|  | **Interpersonal Skills** | 0.230** | 0.272** | 0.320** | 0.173** | 0.262** | 0.234** | 0.221** | 0.286** |
|  | **Constructing Knowledge** | 0.228** | 0.233** | 0.259** | 0.155* | 0.218** | 0.172* | 0.224** | 0.253** |
|  | **Overall Self** | 0.391** | 0.413** | 0.433** | 0.275** | 0.378** | 0.359** | 0.336** | 0.433** |

p-value ** <0.001; * p= 0.01

**Supplementary Table 3.** Correlations between the Nurse Competence Scale (NCS) (29) and the Self Rating Scale of Self-directed Learning (SRSSDL_ITA_) (28) scores: post - pandemic group

|  |  | **NURSE COMPETENCE SCALE** | | | | | | | |
| --- | --- | --- | --- | --- | --- | --- | --- | --- | --- |
|  | **Correlations** | **Helping Role** | **Teaching-Coaching** | **Diagnostic Functions** | **Managing Situation** | **Therapeutic Intervention** | **Ensuring Quality** | **Working Role** | **Overall Competence** |
| **SELF RATING SCALE OF SELF-DIRECTED LEARNING_ITA_** | **Awareness** | 0.245** | 0.271** | 0.189* | 0.160 | 0.238* | 0.241* | 0.235 | 0.252* |
|  | **Attitutes** | 0.247** | 0.306** | 0.247* | 0.255 | 0.266** | 0.256* | 0.289** | 0.301** |
|  | **Motivation** | 0.269** | 0.298** | 0.274** | 0.285 | 0.263** | 0.275** | 0.282** | 0.307** |
|  | **Learning Strategies** | 0.294** | 0.327** | 0.285** | 0.281 | 0.327** | 0.241* | 0.326** | 0.337** |
|  | **Learning Methods** | 0.258 | 0.335** | 0.241* | 0.238 | 0.283** | 0.305** | 0.255* | 0.303** |
|  | **Learning Activities** | 0.119 | 0.129 | 0.098 | 0.109 | 0.109 | 0.150 | 0.144 | 0.137 |
|  | **Interpersonal Skills** | 0.214 | 0.261** | 0.208 | 0.206* | 0.202* | 0.161* | 0.213* | 0.236* |
|  | **Constructing Knowledge** | 0.136 | 0.200* | 0.112 | 0.132 | 0.129 | 0.101 | 0.141 | 0.158 |
|  | **Overall Self** | 0.300** | 0.356 | 0.280 | 0.282** | 0.307** | 0.289** | 0.319** | 0.343** |

p-value ** <0.001; * p= 0.01
